# Clinical Evidence Informing Treatment Guidelines on Repurposed Drugs for Hospitalized Patients During the Early COVID-19 Pandemic: Corticosteroids, Anticoagulants, (Hydroxy)chloroquine

**DOI:** 10.3389/fpubh.2022.804404

**Published:** 2022-02-18

**Authors:** Stefanie Wüstner, Sara Hogger, Daniela Gartner-Freyer, Andrea Lebioda, Katharina Schley, Friedhelm Leverkus

**Affiliations:** ^1^AMS Advanced Medical Services GmbH, Munich, Germany; ^2^Novartis Pharma GmbH, Nuernberg, Germany; ^3^Amgen GmbH, Munich, Germany; ^4^Pfizer Deutschland GmbH, Berlin, Germany

**Keywords:** evidence generation, COVID-19, treatment guidelines, clinical trials, observational studies, RCT, platform trials, repurposed drugs

## Abstract

**Introduction:**

In early 2020, the coronavirus disease 2019 (COVID-19) pandemic spread worldwide, overwhelming hospitals with severely ill patients and posing the urgent need for clinical evidence to guide patient care. First treatment options available were repurposed drugs to fight inflammation, coagulopathy, and viral replication. A vast number of clinical studies were launched globally to test their efficacy and safety. Our analysis describes the development of global evidence on repurposed drugs, in particular corticosteroids, anticoagulants, and (hydroxy)chloroquine in hospitalized COVID-19 patients based on different study types. We track the incorporation of clinical data in international and national treatment guidelines and identify factors that characterize studies and analyses with the greatest impact on treatment recommendations.

**Methods:**

A literature search in MEDLINE was conducted to assess the clinical evidence on treatment with corticosteroids, anticoagulants, and (hydroxy)chloroquine in hospitalized COVID-19 patients during the first year of the pandemic. Adoption of the evidence from this clinical data in treatment guidelines of the World Health Organization (WHO), Germany, and United States (US) was evaluated over time.

**Results:**

We identified 106 studies on corticosteroids, 141 studies on anticoagulants, and 115 studies on (hydroxy)chloroquine. Most studies were retrospective cohort studies; some were randomized clinical trials (RCTs), and a few were platform trials. These studies were compared to studies directly and indirectly referred to in WHO (7 versions), German (5 versions), and US (21 versions) guidelines. We found that initially large, well-adjusted, mainly retrospective cohort studies and ultimately large platform trials or coordinated meta-analyses of RCTs provided best available clinical evidence supporting treatment recommendations.

**Discussion:**

Particularly early in the pandemic, evidence for the efficacy and safety of repurposed drugs was of low quality, since time and scientific rigor seemed to be competing factors. Pandemic preparedness, coordinated efforts, and combined analyses were crucial to generating timely and robust clinical evidence that informed national and international treatment guidelines on corticosteroids, anticoagulants, and (hydroxy)chloroquine. Multi-arm platform trials with master protocols and coordinated meta-analyses proved particularly successful, with researchers joining forces to answer the most pressing questions as quickly as possible.

## Introduction

Two years after the global pandemic began in early 2020, WHO has registered more than 326 million confirmed coronavirus disease 2019 (COVID-19) cases and about 5.5 million deaths due to COVID-19 (1). Given the rapid increase in infections—more than 1 million confirmed COVID-19 cases and more than 60,000 deaths within the first 3 months of the pandemic in 2020 (1)—and the lack of specific treatments for COVID-19—repurposing widely available drugs was the obvious choice in immediate response to the urgent medical need. Drugs that had already proven effective in clinical experience for the treatment of phylogenetically and symptomatically similar diseases, such as severe acute respiratory syndrome (SARS) or Middle East Respiratory Syndrome (MERS) were used (2). There was an urgent need to generate timely evidence for these repurposed treatment approaches with respect to COVID-19 in order to provide trustworthy guidance. A record number of clinical studies, observational and interventional, have been launched worldwide. Since repurposed drugs were readily available to physicians, cohort studies examining various treatment approaches have contributed a large volume of clinical data from patient care. Promising approaches quickly found their way into interventional trials such as RCTs and multi-arm platform trials even though a universal consensus on the most promising candidates was missing (3). Platform trials are a form of RCT often based on a pragmatic master protocol with adaptive features that facilitates collaborative and streamlined efforts to test multiple different treatments in a large patient population while using a single control arm (4).

In the early phase of the pandemic, of particular interest were three groups of repurposed drugs: Firstly, antivirals, such as (hydroxy)chloroquine, an antimalarial drug that affects the endosomal function used by the SARS coronavirus type 2 (SARS-CoV-2) to enter the cell (5). Those were considered to be most effective during the early phase of the COVID-19 disease characterized by rapid virus replication and mild symptoms (6). Secondly, anticoagulants, such as heparin, as thrombosis and coagulopathy seemed to play an important role in the SARS-CoV-2 pathogenesis (7). Thirdly, anti-inflammatory drugs, such as corticosteroids, that counteract the SARS-CoV-2 induced systemic inflammation (8). The late phase of the disease is often characterized by hyperinflammation and acute respiratory distress syndrome (ARDS) in patients with severe illness (6). Corticosteroids, anticoagulants, and (hydroxy)chloroquine exemplify the first repurposed drugs that were considered promising treatment option for patients with COVID-19 during the early pandemic; yet, early hopes did not hold true for all of them.

The translation of research findings from clinical data into medical practice was guided through the development of treatment guidelines published by national and international health authorities and scientific medical societies. Generally, treatment guidelines contain systematically developed statements that reflect the current consensus by an expert panel based on experience and available evidence. Ideally, treatment guidelines also indicate the level of certainty, discuss uncertainties and limitations, and provide clinical data supporting the statements. Since study results were reported frequently and quickly, sometimes as preliminary analysis or preprints, “living” guidelines publishing frequent updates have emerged. However, some repurposed drugs, such as (hydroxy)chloroquine were introduced in general medical practice without strong clinical evidence (9, 10) partly based on political pressure and hype (11). Treatment options included in or excluded from national and international treatment guidelines suggest that sound evidence was available that could be considered by experts and systematic analyses.

We aim to capture the development of global evidence on repurposed drugs, to trace the uptake of this evidence in international and national treatment guidelines for hospitalized COVID-19 patients, and to identify factors that characterize studies and analyses with the greatest impact on treatment recommendations during the first year of the pandemic. From these, we derive future directions on the development of collaborative structures and blueprints for future pandemics.

## Methods

We used two different approaches to identify studies with hospitalized COVID-19 patients receiving corticosteroids, anticoagulants, or (hydroxy)chloroquine and to evaluate the evidence derived from them in the context of treatment guidelines:

1. a literature search to identify the overall body of evidence and
2. studies directly or indirectly referenced in treatment guidelines as an indication for their considerations by the experts.

### Literature Search

A literature search was performed in MEDLINE using “COVID-19” (MEDLINE search filter) and a combination of the terms “corticosteroids,” “anticoagulants,” or “(hydroxy)chloroquine” (see Supplementary Material File 1: Additional Table 1). The search included articles published in English during the first year of the pandemic (from January 1, 2020 to February 28, 2021). Relevant articles were selected by two independent reviewers using eligibility criteria (see Supplementary Material File 1: Additional Table 2) developed according to the PICO scheme (12). Discrepancies between reviewers were dissolved by a consensus-based discussion. Relevant studies enrolled hospitalized adults with COVID-19 that had received corticosteroids, anticoagulants, or (hydroxy)chloroquine, with clinically meaningful endpoints reported, such as mortality, clinical status, hospitalization, or adverse events. Studies with patients receiving the above mentioned drugs for an underlying condition other than COVID-19 were excluded. Since this analysis should provide an overview on evidence generated and published, all study types except case studies were included. When a study reported results for multiple treatments, e.g., a cohort study, those studies were included in the results for each treatment separately.

### COVID-19 Treatment Guidelines

During the first year of the COVID-19 pandemic, treatment guidelines for hospitalized patients evolved as results for numerous observational and interventional studies were made public in the form of press releases, preprints, preliminary analyses, and full study publications. The guidelines provided orientation on possible treatment options and evaluated most recent evidence on treatment of hospitalized COVID-19 patients. We analyzed the uptake of clinical study data over the course of the early pandemic in international (WHO) and national (German and US) guidelines. Therefore, all versions of guidelines for the clinical treatment of hospitalized COVID-19 patients from the WHO, the Association of Scientific Medical Societies in Germany (AWMF)/Germany, and the National Institute of Health (NIH)/US were identified from the beginning of the pandemic to February 28, 2021.

For this analysis, the clinical evidence on patients hospitalized for COVID-19 treated with corticosteroids, anticoagulants, and (hydroxy)chloroquine was extracted from the treatment guidelines over time. Both primary sources directly referred to, such as study publications or preprints, and secondary sources indirectly referred to, such as studies included in systematic reviews and meta-analyses were considered. Studies used to extrapolate clinical information from similar conditions, such as non-COVID-19 ARDS, or related diseases, such as MERS or SARS, were not included.

### Data Extraction and Analyses

Following data was extracted from the eligible studies: (electronic) publication date, study type (platform, RCT, non-RCT interventional, observational), study design (observational studies: prospective/retrospective data collection, matching method, regression analysis; RCTs: blinding), sample size, region, recruitment start and end date, and premature termination. Plots and graphs were produced using R version 4.0.5.

## Results

### Body of Evidence in the Context of Treatment Guidelines

**Figure 1:**
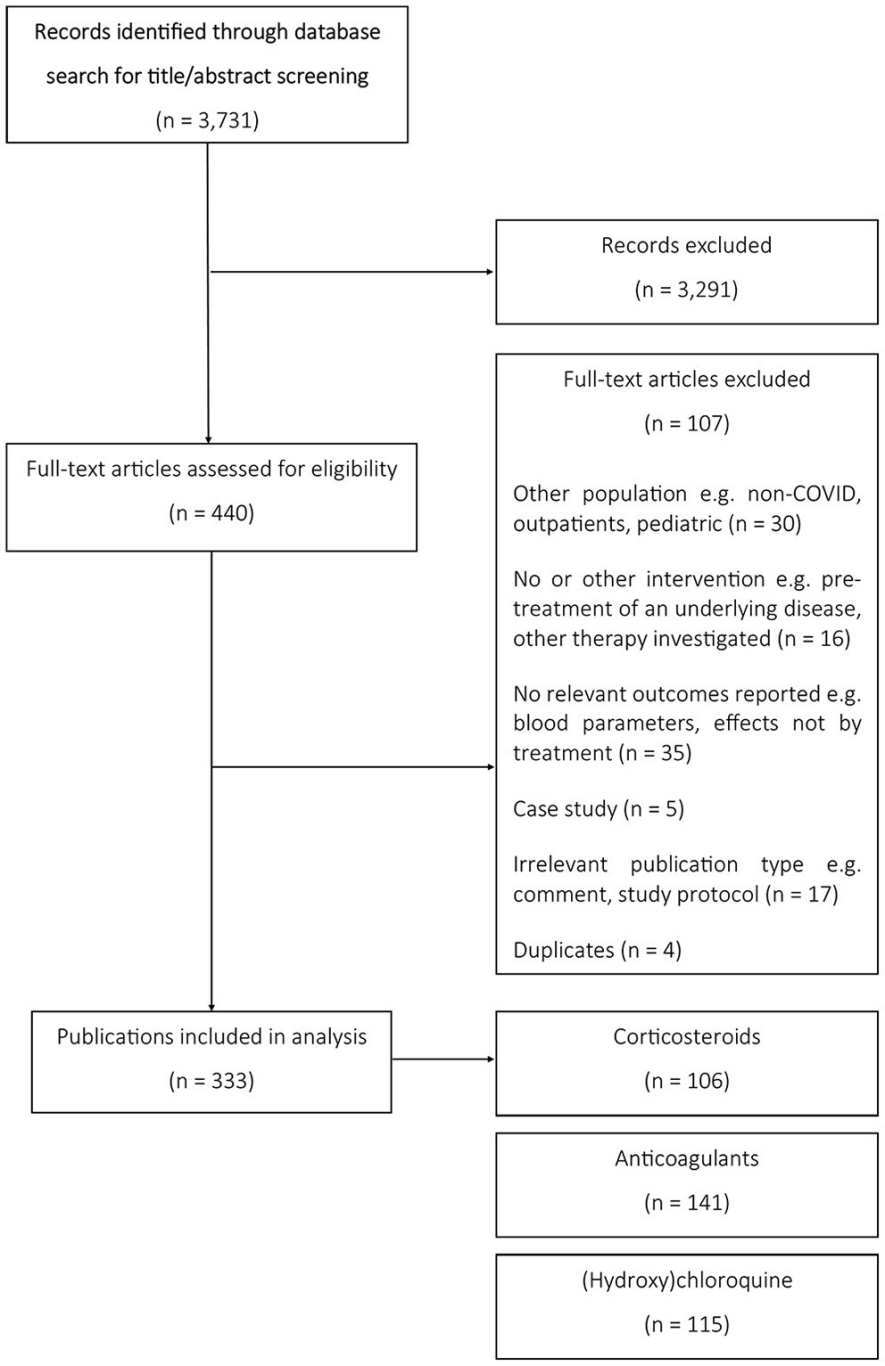
PRISMA flow diagram of search for studies on hospitalized COVID-19 patients.

**Figure 2:**
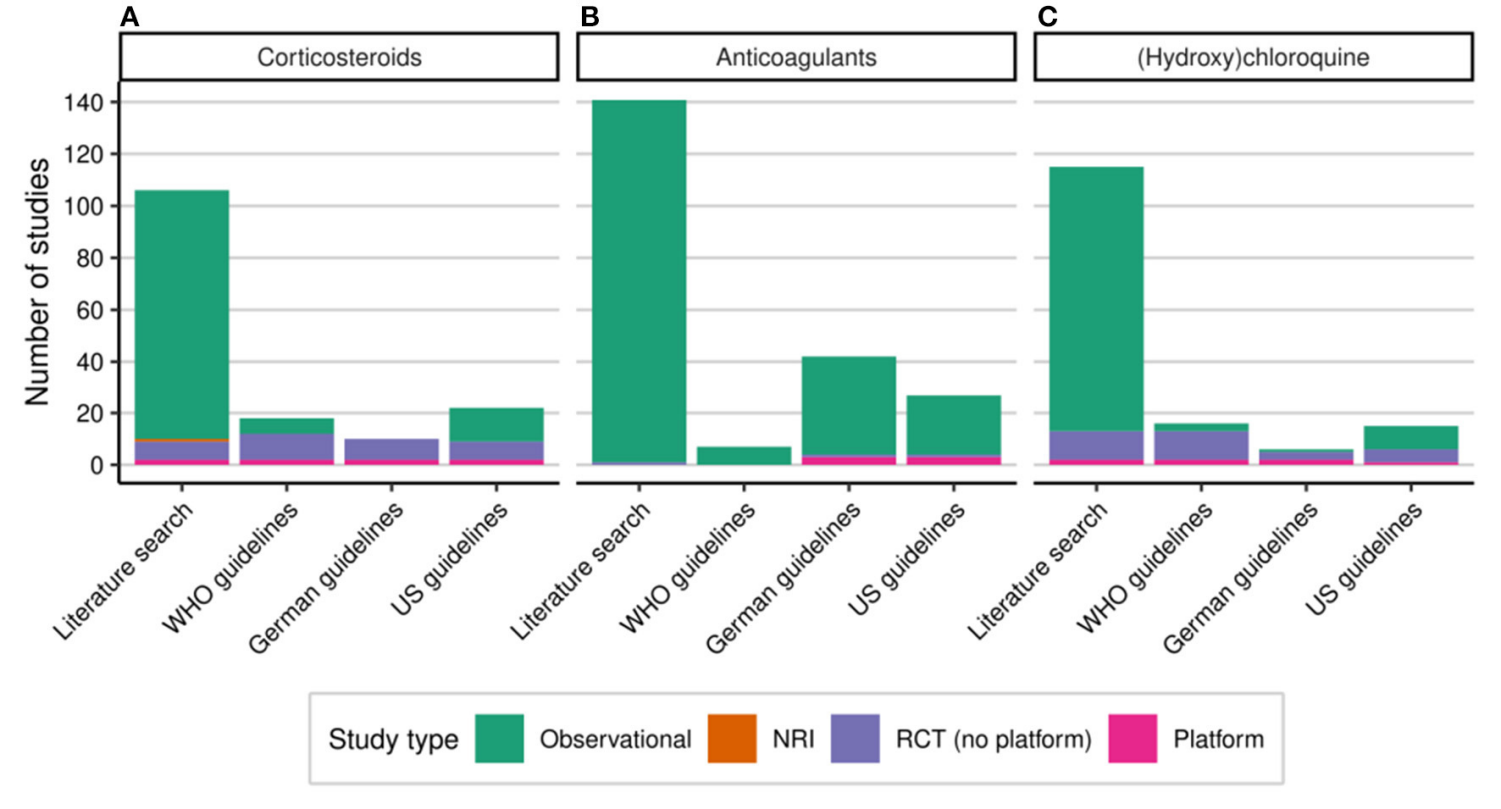
Studies with hospitalized COVID-19 patients treated with corticosteroids **(A)**, anticoagulants **(B)**, or (hydroxy)chloroquine **(C)**. Number of studies identified in (MEDLINE) literature search and directly or indirectly referenced in treatment guidelines up to the end of February 2021 by study type. COVID-19, coronavirus disease 2019; NRI, non-randomized interventional study; US, United States; RCT, randomized controlled study; WHO, World Health Organization.

In our literature search covering the first year of the COVID-19 pandemic, we identified 333 publications reporting results on observational and interventional studies of patients hospitalized for COVID-19 that were treated with corticosteroids (106 studies), anticoagulants (141 studies), and/or (hydroxy)chloroquine (115 studies) (Figure 1). Most studies were observational, in particular cohort studies. We identified seven RCTs and two platform trials for corticosteroids (Figure 2A), only one RCT for anticoagulants (Figure 2B) and 11 RCTs and two platform trials for (hydroxy)chloroquine (Figure 2C). A complete list of studies included in Figure 2 is available in the Supplementary Material (see Supplementary Material File 2: Additional Tables 1–3).

We put this body of evidence formed by observational and interventional studies into the perspective of studies directly or indirectly referred to in treatment guidelines from the WHO, Germany, and US.

The WHO guidelines were developed by a group of clinical content experts, patient -partners, and ethicists. They are a compilation of different types of recommendations: There were three versions of the WHO interim guidance on clinical management of COVID-19 (13), the WHO living guidance on corticosteroids for COVID-19 (14) and two versions of the WHO living guidelines on therapeutics and COVID-19 (15). The WHO has published the first of these six guidelines on March 13, 2020.

The German treatment guidelines were developed by a representative working group of experts from different medical societies led by the German Society of Medical Intensive Care and Emergency Medicine (DGIIN), the German Interdisciplinary Association for Intensive Care and Emergency Medicine (DIVI), the German Respiratory Society (DGP), and the German Society of Infectious Diseases (DGI). The guidelines were classified according to the process of consensus building and to evidence retrieval and synthesis: S1 (expert recommendations; informal consensus), S2k (consensus-based), and S3 (evidence- and consensus-based; systematic search) type guidelines (16). In the period under review, the German working group published three versions of the S1 guideline for patients with COVID-19 in intensive care (17–19), a S2k guideline (20), and a S3 guideline (21). The latter two extending the recommendations from the intensive care unit (ICU)-setting to all hospitalized patients. The first of these five guidelines has been published on March 12, 2020.

The US guidelines were developed by a panel composed of representatives from federal agencies, health care organizations, and academic institutions as well as professional societies that have expertise in the relevant field in order to provide the most recent information on optimal management of COVID-19. Each statement is evaluated in terms of recommendation level (strong, moderate, optional) and evidence quality (I, II, III). The NIH published the first US guideline on COVID-19 treatment on April 21, 2020, followed by 20 updates in the period of interest (22); thus, a total of 21 versions of US guidelines were available for the analysis.

Not surprisingly, we found that when RCTs were available they were referred to in treatment guidelines at a much higher proportion than observational studies (Figures 2A–C). Nevertheless, a substantial amount of observational studies were considered in the treatment guidelines, in particular when robust evidence from RCTs was missing. The clinical evidence from different study types and the way it impacted treatment guidelines over time is described in the following sections for the three different treatment options.

### Corticosteroids

For corticosteroids, we identified a body of clinical evidence consisting of 106 eligible studies in hospitalized COVID-19 patients in our literature search (Figure 2A). Of those, 96 studies were observational, one study was interventional without randomization, and nine studies were RCTs (including two platform trials). The extraction of relevant studies from all versions of the WHO, German, and US guidelines until February 28, 2021 resulted in 18, 10, and 22 studies, respectively (Figure 2A). The WHO and US guidelines referred also to observational studies while the German guidelines only included RCTs and platform trials. In the following sections, we describe the overall contribution different studies on corticosteroids with respect to study type and patient number as well as the incorporation of evidence in treatment guidelines over time.

The observational studies identified in our literature search were predominantly retrospective cohort studies (79.2%). Of the remaining studies, 9.4% were prospective cohort studies, 8.3% did not report whether patients were observed prospectively or retrospectively, two studies were before-and-after studies and one study was a retrospective case-control-study. The majority of observational studies (85.4%) used statistical methods to control confounding, such as multivariate models, propensity score methods, or a before-and-after design. These methods can reduce confounding but cannot rule out all biases since not all confounding factors may be known or assessed. Overall, these studies were very heterogeneous with respect to their design, intervention, analysis, outcome, and reporting. Only a few of those were explicitly considered in the guidelines, as described below.

From the nine RCTs (including two platform trials) identified in our literature search, eight were also included in the treatment guidelines (23–30). Conversely, of 12 RCTs included in at least one of the guidelines, four RCTs were preprints (31) or studies that have not been published yet [DEXA-COVID, COVID STEROID, Steroids-SARI, from (32)], so they were not covered by our literature search. The two platform trials RECOVERY (28) and REMAP-CAP (33) were included in our literature search and all guidelines analyzed. Altogether, the literature search and the guidelines resulted in 13 RCTs, of these five were blinded (25, 27, 29, 31) [COVID STEROID, unpublished, from (32)], one was single-blinded (30) and seven were not blinded (24, 26, 28, 33, 34) [DEXA-COVID and Steroids-SARI, unpublished, from (32)]. Blinding of RCTs with repurposed drugs can be difficult due to lack of placebo control. However, risk of bias can be minimized by “hard” endpoints such as mortality. Four RCTs were stopped early, either because of decreased number of COVID-19 cases (30) or because the results of the RECOVERY platform trial were published (25, 26, 35). After the RECOVERY platform trial showed a clear benefit for COVID-19 patients receiving corticosteroids in day-28 mortality, it was no longer considered ethical to enroll patients in these trials.

**Figure 3:**
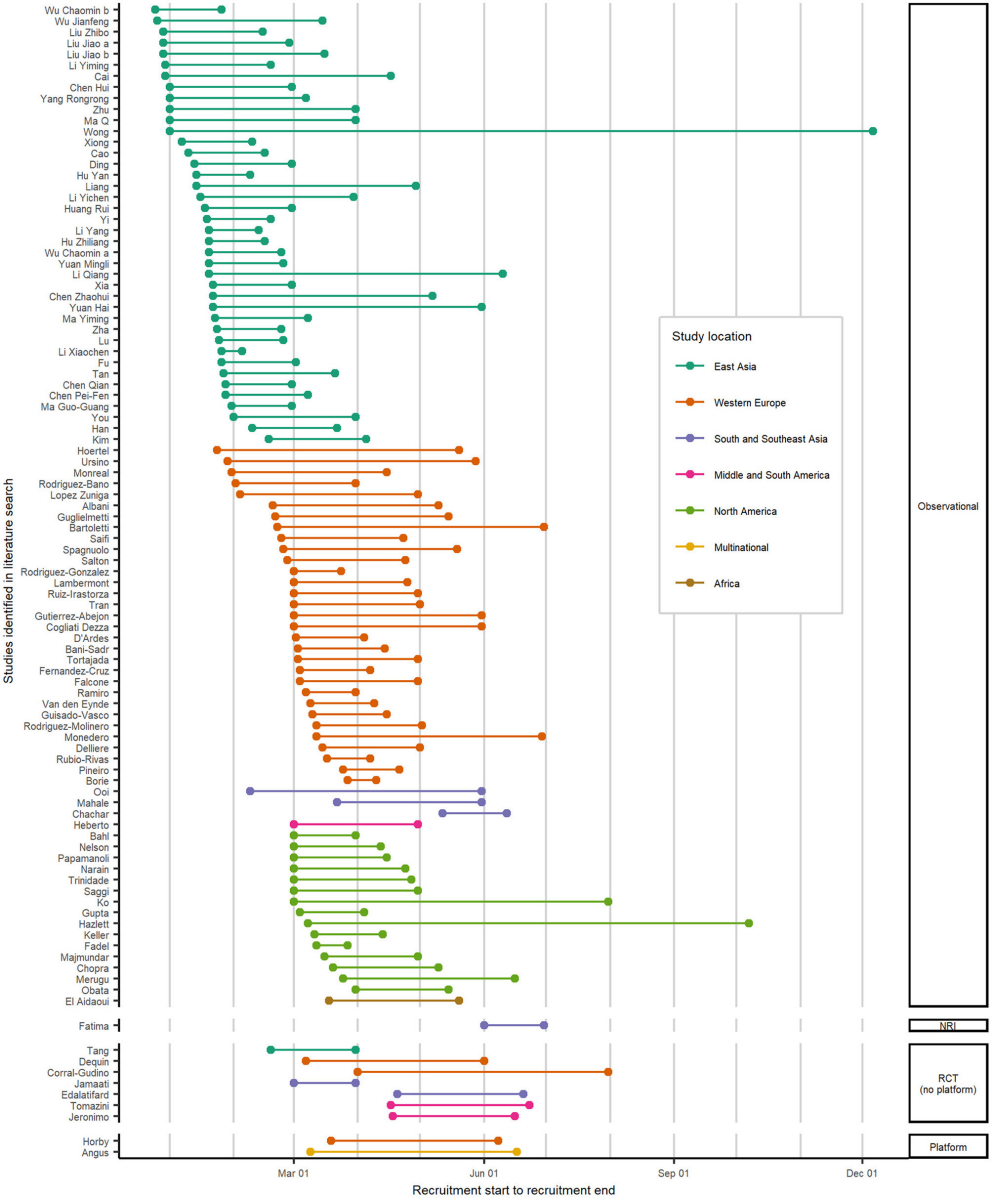
Spatial and temporal distribution of studies with hospitalized COVID-19 patients treated with corticosteroids in 2020. Recruitment start and end dates by geographical regions and grouped by study type for studies identified in the literature search. The lines indicate the period during which patients were enrolled for each study. For five observational studies, the recruitment time was not reported, so these are not included in this figure. COVID-19, coronavirus disease 2019; NRI, non-randomized interventional study; RCT, randomized controlled study.

Taking a closer look at the studies reporting results on patients treated with corticosteroids in 2020, we see a chronological progression of these studies regarding the study type and the region (Figure 3). The spatial and temporal distribution of observational studies primarily reflects the surge in cases as the disease spread worldwide. The first patients to be evaluated as part of an observational study were treated with corticosteroids in December 2019 in China (East Asia), followed by patients from France and Spain (Western Europe) in January 2020. From February 2020, patients in Singapore (South and Southeast Asia), Korea (East Asia), and Italy (Western Europe); and from March 2020, patients in Iran and India (South and Southeast Asia), in the USA and Mexico (North and Middle America), and patients in further Western European countries were enrolled in observational studies.

The first RCT started recruitment in February 2020 in China (30). In March and April 2020, 6 more RCTs started in Iran, France, Spain, Brazil, and Iran. The two platform trials RECOVERY (28) and REMAP-CAP (33) enrolled patients treated with corticosteroids between March and June 2020.

In summary, while lots of observational studies on corticosteroids were conducted worldwide with focus on East Asia, especially China, and Western Europe, throughout the first year of the pandemic, only a handful RCTs were carried out, and only one RCT, the REMAP-CAP platform trial (23), was multinational.

**Figure 4:**
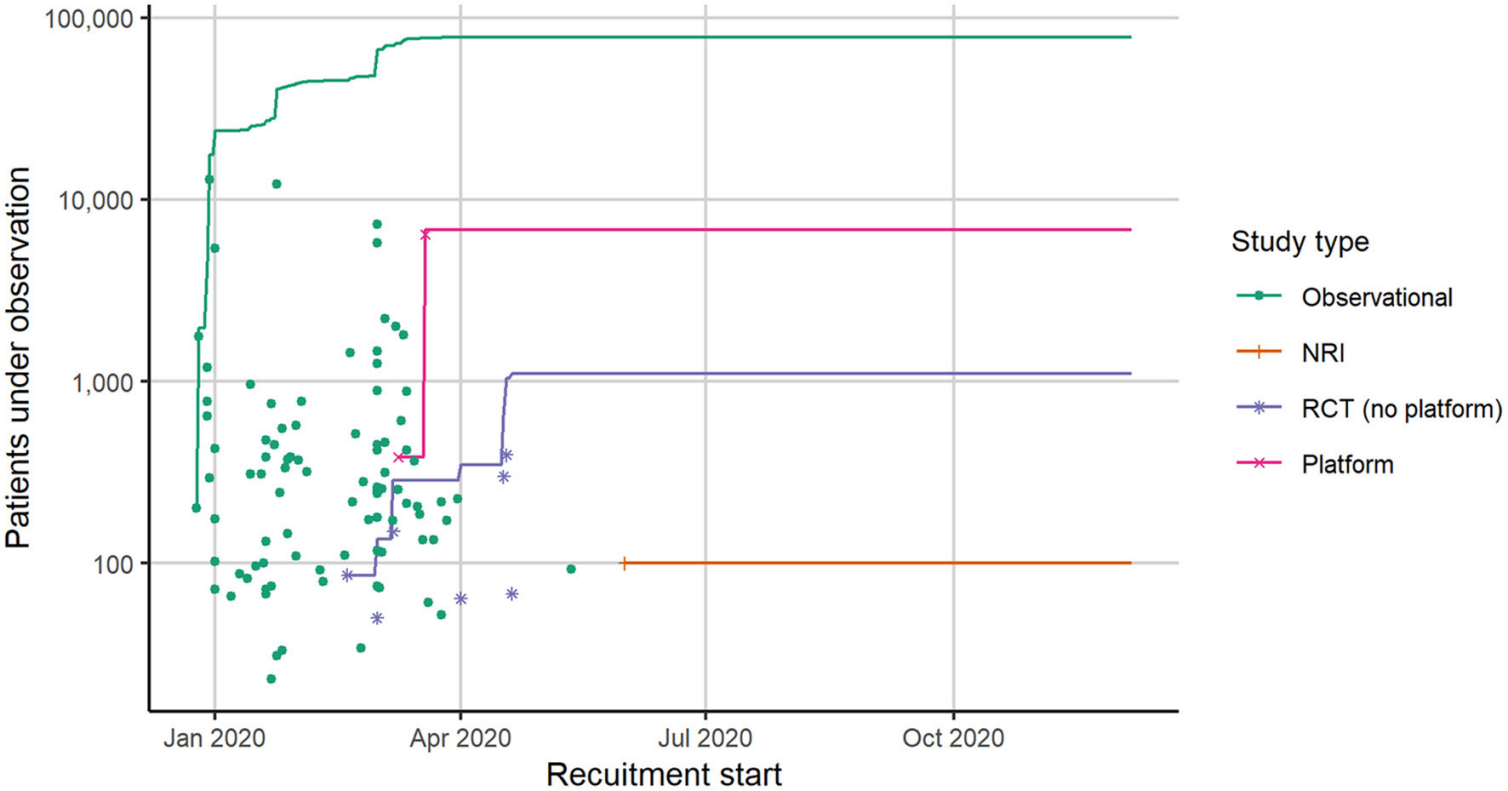
Number of hospitalized COVID-19 patients in studies identified in literature search for corticosteroids. Each dot represents the recruitment start date and the number of cases included in a single study. The lines represent the number of cumulative cases under observation across all studies per study type. For five observational studies, the recruitment start date was not reported, so these are not included in this figure. COVID-19, coronavirus disease 2019; NRI, Non-randomized interventional study; RCT, randomized controlled study.

We also analyzed the number of patients with COVID-19 enrolled in studies on corticosteroids by study type (Figure 4). Figure 4 shows the number of patients enrolled and the recruitment start date for the studies identified in the literature search (studies as in Figure 2A). The sample sizes in observational studies varied widely from 23 to nearly 13,000 patients per study. Most patients were enrolled within the first 5 months of the pandemic. During the first year of the COVID-19 pandemic, nearly 100,000 patients were followed within observational studies to evaluate, the effect of corticosteroids, partly among other treatments. Four RCTs had a sample size of less than 100 patients (24, 29, 30, 34), the other three RCTs had a sample size of approx. 150–400 patients (25–27). Within the two multi-arm platform trials, 6,425 [RECOVERY (28)] and 384 [REMAP-CAP (33)] patients were randomized to corticosteroids or control.

The cumulated sample size by study type shows differences between the study pool from the literature search and from the treatment guidelines (studies that are included in Figure 2A). On the one hand, summing up all patients from studies identified in our literature search, only 9.2% were enrolled in RCTs (including platform trials), while 90.1% were enrolled in observational studies. On the other hand, summing up all patients from studies included in treatment guidelines, 56.6% were enrolled in RCTs (including platform trials) and 43.4% were enrolled in observational studies. Remarkably, the RECOVERY platform trial included almost four times more patients than all other RCTs together.

That means, patient enrolled in RCTs and especially in platform trials contributed disproportionately more to evidence-based decision-making than patients observed in observational studies. Thus, the mere volume of patients under observation is not the decisive factor in generating robust evidence.

Treatment guidelines on corticosteroids were evolving as more evidence became available. In the beginning of the COVID-19 pandemic, the potential benefits and harms of corticosteroids for patients with COVID-19 were controversial. Results from clinical studies regarding corticosteroid treatment of the hyperinflammatory state in non-COVID ARDS (e.g., from SARS-CoV, MERS-CoV, influenza) were inconclusive (36–38). Early observational studies from December 2019 to March 2020 (Figure 3) resulted in different conclusions with respect to efficacy and safety of corticosteroids with some cohorts reporting negative [e.g., (39, 40)] and others positive effects [e.g., (41)].

Table 1 gives an overview of the studies referred to in the respective versions of the WHO, German, and US guidelines. Guideline versions in which changes were made with respect to the underlying evidence or recommendations are presented.

**Table 1:** Studies directly or indirectly contributing evidence to treatment guidelines on corticosteroids.

**Study name First Author publication date (epub date if available)**	**Publication type**	**WHO guidelines**				**German guidelines**				**US guidelines**					
		**27-May-20**	**02-Sep-20**	**20-Nov-20**	**17-Dez-20**	**16-Jun-20**	**21-Jul-20**	**23-Nov-20**	**23-Feb-21**	**21-Apr-20**	**25-Jun-20**	**17-Jul-20**	**30-Jul 20**	**27-Aug-20**	**03-Nov-20**
**Huang** 24-Jan-20	Study										(O)^1^	(O)^1^	(O)^1^	(O)^1^	(O)^1^
**Yang** 24-Feb-20	Study	(O)^2^													
**Guan** 28-Feb-20	Study	(O)^2^													
**Zhou** 9-Mar-20	Study	(O)^2^													
**Wang** 12-Mar-20	Preprint									O	(O)^1, 3^	(O) ^1, 3^	(O)^1, 3^	(O)^1, 3^	(O)^1, 3^
**Wu** 13-Mar-20	Study	(O)^2^								O^3^	O^3^	O^3^	O^3^	O^3^	O^3^
**Sun** 17-Mar-20	Preprint									O					
**Lu** 11-Apr-20	Preprint		(O)^4^		(O)^4^										
19-May-20	Study											O	O	O	O
**Li** 12-Apr-20	Study		(O)^4^		(O)^4^										
**Wang** 28-Apr-20	Letter												O	O	O
**Fadel** 19-May-20	Study										O	O	O	O	O
**Yuan** 2-Jun-20	Study										O	O	O	O	
**Fernandez-Cruz** 22-Jun-20	Study												O	O	O
**Keller** 22-Jul-20	Brief report												O	O	O
**Nelson** 9-Aug-20	Study													O	O
**Li** 2-Sep-20	Study														O
**Salton** 12-Sep-20	Study														O
**GLUCOCOVID Corral** 18-Jun-20	Preprint		R	R^5^	R^5^			(R)^6^	R		R	R	R	R	R
**MetCOVID Jeronimo** 12-Aug-20	Study		R7		R^5, 7^			R^6, 7^	R						R^7^
**CAPE COVID Dequin** 2-Sep-20	Study		R^7^	R	R^5, 7^			R^6, 7^	R						R^7^
**CoDEX Tomazini** 2-Sep-20	Study		R^7^	R	R^5, 7^			R^6, 7^	R						R^7^
**COVID STEROID** 2-Sep-20	Meta-analysis		(R)^7^		(R)^7^			(R)^7^							(R)^7^
**DEXA-COVID** 2-Sep-20	Meta-analysis		(R)^7^		(R)^7^			(R)^7^							(R)^7^
**Steroids-SARI** 2-Sep-20	Meta-analysis		(R)^7^		(R)^7^			(R)^7^							(R)^7^
**Farahani** 9-Sep-20	Preprint				(R)^7^										
**Edalatifard** 17-Sep-20	Study				(R)^7^				R						
**RECOVERY Horby** 16-Jun-20	Press release					P					P	P	P	P	P
22-Jun-20	Preprint		P	(P)^5^	(P)^5^			(P)^6^			P	P	P	P	P
17-Jul-20	Study		P^7^	P^5^	P^5, 7^		P	P^6, 7^	P					P	P^7^
**REMAP-CAP Angus** 2-Sep-20	Study		P^7^	P	P^5, 7^			P^6, 7^	P						P^7^
Mild—moderate		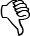	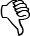	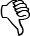	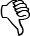	–	–	–	–	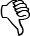	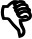	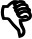	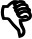	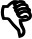	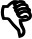
Severe—critical		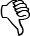	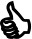	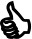	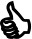	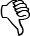	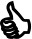	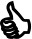	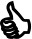	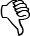	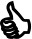	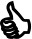	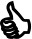	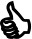	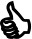

*1, (42); 2, (36); 3, (37); 4, (43); 5, 3^rd^ Version (Update 2) (44); 6, (14); 7, (32)*.

*All guideline versions that include further evidence on the clinical benefit or safety of corticosteroids compared to the previous version are listed. Studies directly and/or indirectly (brackets) referenced. Secondary sources according to footnotes. Treatment guidelines for patients with mild—moderate and severe—critical illness were abstracted together into “thumbs down” for recommendation against, “thumbs up” for recommendation for (with restrictions) and “–” when this patient group is not mentioned. O, observational; R, RCT; P, Platform trial*.

The first WHO (13) and German (17) guidelines from March 2020 recommended against routine use of steroids for patient with COVID-19 viral pneumonia and ARDS, respectively, due to lack of efficacy data and previously observed side effects (e.g., hyperglycemia, secondary infections, reactivation of latent infections, delayed viral clearance) from indirect evidence (45–47). Likewise, the first version of the US guideline, published April 21, 2020, recommended against the routine use of systemic corticosteroid for hospitalized patients with COVID-19 that are non-critically ill or mechanically ventilated without ARDS (22). For critically ill patients with ARDS that are mechanically ventilated, the US guideline stated that there are insufficient data to support a recommendation for or against treatment with corticosteroids.

The US guideline was the first guideline that referred to clinical data on corticosteroids from COVID-19 patients (Table 1). The authors alluded to cohort studies from China that reported that methylprednisolone might be beneficial for patients with COVID-19 considering symptom resolution and mortality, yet cautioned with respect to limitations such as lack of control, small sample size, and lack of information on exact dose and timing (48–50). Two of those studies were only published as preprint (49, 50) and one was already published ahead of print (48). The results from the latter a retrospective cohort study of COVID-19 patients (*n* = 201) by Wu et al. were stated in the rationale. This study showed an association between methylprednisolone therapy and lower mortality [hazard ratio (HR) 0.38; 95% CI 0.20–0.72] (48). It was criticized that the analysis was not adjusted for confounding factors, such as confounding by indication (22) April 21, 2020.

In May 2020, the WHO guideline (13) also referred to evidence on corticosteroid treatment from observational studies citing a systematic review that meta-analyzed cohorts of patients with SARS-CoV-2, SARS-CoV, and MERS-CoV infections (36). This analysis included Wu et al. and three different cohort studies (Table 1). The recommendation against the routine use of systemic corticosteroids for treating viral pneumonia remained unchanged (13).

On June 16, 2020, the University of Oxford reported results from RECOVERY platform trial in a press release. This large, open-label, multi-arm RCT showed a statistically significant survival benefit of patients treated with low-dose dexamethasone (*n* = 2,104) that were mechanically ventilated (HR 0.64; 95% CI 0.51–0.81) or receiving oxygen (HR 0.82; 95% CI 0.72–0.94) compared to those that received standard of care (*n* = 4,321) (51). On the same day, this information was introduced in the background texts of the German S1 guideline (18). This indicates that this study was already seen as a breakthrough in clinical management of critically ill patients.

The preprint of the study publication was available on June 22, 2020 (52). Only 3 days later, the US guideline recommended using dexamethasone for those patients, that profited in the RECOVERY platform trial and against using it in patients that do not require supplemental oxygen (22) June 25, 2020. Furthermore, the recommendation on corticosteroids was extended to the use of alternative glucocorticoids, such as methylprednisolone or hydrocortisone (22) July 30, 2020. This recommendation based on expert opinion was substantiated by referencing reports of several cohort studies and a non-peer reviewed RCT (GLUCOCOVID) with 85 patients (Table 1).

Within a month, on July 17, 2020, the results from the RECOVERY platform trial were published in a peer-reviewed prestigious journal ahead of print (28). Shortly thereafter, German guidelines fully adopted results as a basis for their recommendation to treat COVID-19 patients that need to be ventilated with low-dose dexamethasone (19).

The WHO published their recommendation for corticosteroids (14) together with a meta-analysis by the WHO Rapid Evidence Appraisal for COVID-19 Therapies (REACT) Working Group (32). For this meta-analysis, the WHO invited investigators that had registered RCTs on corticosteroids in patients with COVID-19. Together they developed the protocol as well as coordinated analyses, data-cuts, and publications. The meta-analysis included seven RCTs: RECOVERY (28), MetCOVID (27), CAPE COVID (25), CoDEX (26), REMAP-CAP (23), DEXA-COVID [unpublished, from (32)], and Steroids-SARI [unpublished, from (32)]. Thereby, also studies stopped due to the results of the RECOVERY trial could contribute their data. The meta-analysis showed a positive survival effect for critically ill patients, which supported the use of corticosteroids.

The platform trials RECOVERY and REMAP-CAP, along with other RCTs that have been meta-analyzed were referenced in all subsequent WHO, German, and US guideline versions. The results of these platform trials have since formed the basis of the treatment guidelines for corticosteroids (Table 1).

### Anticoagulation

For anticoagulants, we identified 141 eligible studies in hospitalized COVID-19 patients in our literature search (Figure 2B). Almost all studies (140/141) were observational. Most observational studies were retrospective cohort studies and a substantial proportion were only published as letters. The extraction of relevant studies from all versions of the WHO, German, and US treatment guidelines led to 7, 42, and 27 studies, respectively (Figure 2B). The difference between the numbers of studies in the guidelines is due to indirect references from meta-analyses and clinical guidance documents. The WHO guidelines considered a clinical guidance conducted by ASH with six observational studies (53) and the German guidelines included a pooled analysis published by Patell et al. containing 31 relevant observational studies (54). The US guidelines referenced several observational studies directly but also included a number of clinical guidance documents with observational studies included [e.g., (55–57)].

Only one RCT published by Lemos et al. with only 20 patients (58) was identified within the literature search, this RCT was also referred to in the German and US, but not in the WHO guidelines. Additionally, the German and US guidelines included very preliminary data on the joint interim analysis of three platform trials ATTACC, ACTIV-4a, and REMAP-CAP (59, 60).

In the very beginning of the COVID-19 pandemic, the WHO guideline recommended that patients with critical illness should receive pharmacological prophylaxis for prevention of venous thromboembolism (VTE) preferably with low molecular-weight heparin, if no contraindications exist, based on indirect evidence of patients in the ICU (13) March 13, 2020]. German and US guidelines did not mention anticoagulation in the first versions (17) March 12, 2020 and (22) April 21, 2020, respectively.

Within the first months of the pandemic, it became apparent that COVID-19 is associated with an increased incidence of thrombotic and thromboembolic events. Patients with COVID-19 in intensive care receiving a standard VTE prophylaxis still had a high incidence of thrombotic complications correlated with an increased D-dimer level (61). A cohort from China found that the mortality of patient with severe COVID-19 was reduced in patients receiving VTE prophylaxis compared to those who did not receive anticoagulants (62).

In May 2020, a section on antithrombotic therapy in patients with COVID-19 was included in the US guideline (22). It stated that all hospitalized patient with COVID-19 should receive standard VTE prophylaxis. This recommendation was accompanied by evidence from cohort studies that had high incidences of VTE in patients in or admitted to the ICU despite prophylactic anticoagulation (61, 63–66). Nevertheless, the US guidelines stated that anticoagulant doses for VTE prophylaxis should only be increased in the setting of a clinical study (22) May 12, 2020.

Additionally, the US guideline introduced a retrospective cohort study of 2,773 patients in New York, published by Paranjpe et al. as letter, where among the subpopulation of 395 mechanically ventilated patients only 29.1% of patients receiving therapeutic anticoagulation died in hospital, while 62.7% of patients not receiving anticoagulation died [adjusted HR (aHR) of 0.86; 95% CI 0.82–0.89] (67). This effect was not seen for the overall cohort. Due to limitation such as lack of detailed patient characteristics, reasons for initiation of anticoagulant therapy, and the potential impact of survival bias, these results did not influence the treatment recommendations (22) May 12, 2020.

In June 2020, the German guideline recommended, that all hospitalized patients with COVID-19 should receive a VTE prophylaxis with using a dose approved for a high-risk of VTE, based on expert consensus (18). The rationale mentioned is, that in ICU patients a standard VTE prophylaxis is not sufficiently effective as seen in observational studies. Therefore, intensified anticoagulation should be considered in ICU patients. However, the use of therapeutic anticoagulation was not routinely recommended without diagnosis of VTE or extracorporeal membrane oxygenation (ECMO), yet, seemed to be justifiable on a case-to-case basis.

In October 2020, a retrospective analysis of 4,389 patients from New York, published online by Nadkarni et al., found that prophylactic as well as therapeutic anticoagulation were associates with reduced in-hospital mortality compared to no anticoagulation (aHR 0.50; 95% CI 0.45–0.57 and aHR 0.53; 95% CI 0.45–0.62, respectively) (68). The difference between therapeutic and prophylactic anticoagulation was not statistically significant. Bleeding rates were higher in patients on therapeutic anticoagulation (3.0%) compared to patients on prophylactic (1.7%) or no anticoagulation (1.9%). To correct for potential confounding, inverse probability of treatment weights (IPTW) models were used. Additionally, estimates were adjusted by multinomial logistic model for multiple predictors, such as age, sex, ethnicity, body mass index, and prior use of anticoagulants.

The New York cohort study by Nadkarni et al. was also introduced in the text of the next version of the German guideline on treatment of hospitalized patients with COVID-19 in November 2020 (20). This version was updated to a higher methodological quality level, which requires formal consensus (S2k). The guideline strongly recommended that all hospitalized patients receive standard pharmacological thromboprophylaxis, if not contraindicated. It stated that those patients with additional risk factors favoring VTE, such as obesity and ICU treatment, and low risk of bleeding can receive an intensified thromboprophylaxis.

Results on mortality from the New York cohort study by Nadkarni et al. based on evidence from a living review provided by the American Society of Hematology (ASH) (53) have been included by WHO in their considerations (13) January 25, 2021. For hospitalized patients without an indication for therapeutic anticoagulation, the WHO recommended standard thromboprophylaxis rather than therapeutic or intermediate-dose anticoagulation. They concluded that therapeutic or intermediate-dose anticoagulation can possibly, with very low certainty, reduce mortality (aHR 0.86; 95% CI 0.73–1.02) (68) and pulmonary embolism [odds ratio (OR) 0.09; 95% CI 0.02–0.57] (69) but that the risk of major bleeding is probably increased [OR 1.42 (matched case control) (70) to OR 3.89 (retrospective cohort) (71)]. The risks that were also supported by indirect evidence from RCTs of therapeutic anticoagulation for other indications were rated higher that potential benefits observed in observational studies in patients with COVID-19.

The chapter on antithrombotic therapy in patients with COVID-19 of the US guideline was updated in December 2020 but recommendations from May 2020 remained unchanged: All hospitalized patients with COVID-19, including critically ill patients, should be treated with prophylactic dose anticoagulation (22). The text referred to the analysis performed by ASH, but not as the WHO on those with acute illness, but with critical illness (55). In this analysis, a cohort of 141 critically ill patients from three hospitals in Colorado by Ferguson et al. was included for mortality instead of the New York cohort by Nadkarni et al. The mortality in patients who received therapeutic anticoagulation vs. those who received a prophylactic dose did not differ (OR 0.73; 95% CI 0.33–1.76) (72). Additionally, the guideline reported the results from a smaller New York cohort by Paranjpe et al. (67), as before, and a small RCT by Lemos et al. with 20 mechanically ventilated patients treated with either therapeutic or prophylactic anticoagulation (58).

In December 2020 and January 2021, the prospective multiplatform of three randomized, adaptive, open-label platform trials, namely ATTACC, ACTIV-4a, and REMAP-CAP, published press releases and presented preliminary data on their website on a planned interim analysis based on a Bayesian approach comparing prophylactic and therapeutic anticoagulation (59, 60, 73). In this analysis, 1,123 patients with moderate disease who were hospitalized but not admitted to the ICU appeared to benefit from therapeutic anticoagulation vs. prophylactic anticoagulation, so this arm was stopped for superiority. On the contrary, therapeutic anticoagulation appeared to pose a risk for critically ill patients in the ICU compared to prophylactic anticoagulation; as an interim analysis of 1,205 patients with severe COVID-19 showed that predefined criteria for futility were met, enrollment in this part of the study was halted.

In February 2021, a reference to these very preliminary results was provided in the German S3 guideline in the context of therapeutic anticoagulation (21) as well as in the US guideline (22). However, these results were not yet considered for their recommendations.

The German S3 guideline stated that the recommendation for optional use of intensified thromboprophylaxis in hospitalized COVID-19 patients with additional risk factors for VTE is based on expert opinion and observational studies that have been systematically reviewed in a pooled analysis (54).

Pharmacologic prophylactic and therapeutic anticoagulation of hospitalized COVID-19 patients to prevent VTE serve as an example where multiple cohort studies played a major role in shaping expert opinions and supporting treatment guidelines (Figure 2B). Even though the importance of thromboprophylaxis was widely accepted and put into practice, the large amount of observational data was not sufficient, to guide clinicians to choose the right intensity of anticoagulation considering the patient's risk of thrombosis and bleeding in the context of COVID-19. High-quality evidence from RCTs and platform trials comparing different types and intensities of anticoagulation was eagerly awaited because the indication for intensified or therapeutic anticoagulation in hospitalized patients with COVID-19 was still not well-defined.

### (Hydroxy)Chloroquine

For (hydroxy)chloroquine, we identified a body of clinical evidence consisting of 115 eligible studies in our literature search (Figure 2C). Overall, 102 studies (88.7%) were observational. Of these, 26.1% were mainly dealing with the effect of (hydroxy)chloroquine ± azithromycin on QTc prolongation, an adverse drug reaction that can predispose patients for potentially fatal cardiac arrhythmias. Furthermore, 13 RCTs including two platform trials, RECOVERY and SOLIDARITY, were found. The extraction of relevant studies from all versions of the WHO, German, and US guidelines resulted in 16, 6, and 15 studies, respectively (Figure 2C). While the WHO and German guidelines mostly referred to RCTs and platform trials, the US guidelines also included direct evidence from observational studies, in particular at a time, when mainly retrospective cohort studies were available. The overlap between our literature search and the studies included in the treatment guidelines consists of eight observational studies and 10 RCTs, including the two platform trials RECOVERY and SOLIDARITY. Three RCTs that were included in at least one of the guidelines were not covered by our search as they were preprints (74, 75) or only available in Chinese language (76). Conversely, three RCTs that we found in our literature search were not included in the guidelines (77–79) presumably, because they did not contribute additional evidence and were not yet included in a meta-analysis.

Of the many published studies on (hydroxy)chloroquine, there have only been a handful of high-quality landmark studies that have impacted the treatment recommendations or their level of certainty. These include one large observational study by Geleris et al. (80), one small RCT, CloroCOVID-19 (81), as well as two platform trials, RECOVERY (82) and SOLIDARITY (83). Additionally, the WHO supported its strong recommendation against (hydroxy)chloroquine with a network meta-analysis that included 30 studies on (hydroxy)chloroquine in hospitalized patients (44).

In their first treatment guideline in March, 2020, the WHO did not mention (hydroxy)chloroquine as potential treatment option (13), while the German guideline for patients in intensive care pointed out that it is one of the substances under clinical investigation that might possibly be used on a case-to-case basis considering the benefit-risk-ratio without referring to clinical data (17). In its first version, the US guideline referred to a mix of low-quality observational studies and RCTs published only as preprints, letters, or in Chinese only and concluded that further studies are required (22). They strongly recommended against the use of a combination of (hydroxy)chloroquine and azithromycin outside of clinical studies due to a risk of QTc prolongation based on expert opinion.

In April 2020, a trial from Brazil, CloroCOVID-19, comparing two doses of chloroquine was stopped after enrolling 81 patients, since they observed a trend toward a higher mortality in the group with the higher dose (81). The authors state, that it did not seem ethical to randomize to placebo since chloroquine was the local standard of care at the time. Based on this study the NIH changed its US guideline recommending against using high-dose chloroquine for the treatment of COVID-19 (22) May 12, 2020] and the WHO recommended that chloroquine and hydroxychloroquine should not be administered as treatment outside of clinical studies (13) May 27, 2020.

In May 2020, a large cohort study of 1,446 patients with COVID-19 in New York was analyzed by Geleris et al. showing no beneficial of hydroxychloroquine with respect to mortality or need of mechanical ventilation (80). Considering this study and other case series, the panel of the US guideline recommended against the use of (hydroxy)chloroquine for the treatment of COVID-19, except in a clinical study (22) June 16, 2020.

Potential benefits and risks derived from the CloroCOVID-19 trial and the New York cohort study by Geleris et al. were also considered in the second version of the German guideline that recommended that (hydroxy)chloroquine should only be used in clinical studies (18).

Interestingly, the US and the German guideline also referred to another RCT with 62 hospitalized patients from Wuhan which was only published as preprint (75) indicating the desperate need for high-quality evidence.

The RECOVERY platform trial study, which also delivered strong evidence for corticosteroids, was crucial for the recommendations on (hydroxy)chloroquine therapy. In this study, 1,561 patients were randomized to receive hydroxychloroquine and 3,155 patients to receive standard of care. The mortality between the two arms were not significantly different (HR 1.09; 95% CI 0.96–1.23) (82). This resulted in many RCTs being stopped or not even starting recruitment.

The US guidelines were changed based on data from the RECOVERY platform trial published as preprint, recommending that chloroquine or hydroxychloroquine should not be used for hospitalized patients with COVID-19 (22) August 27, 2020. The German S2k guideline did put forward a similar recommendation once results from the RECOVERY platform trial were published in a peer-reviewed journal (20).

The evidence on (hydroxy)chloroquine further solidified by results from the multi-national multi-arm SOLIDARITY platform trial. In this study, 954 patients were randomized to receive hydroxychloroquine and 4,088 patients to control. The in-hospital mortality did not differ significantly between patients treated with hydroxychloroquine and their control (HR 1.19; 95% CI 0.89–1.59) (83).

In December 2020, the WHO issued a strong recommendation against the use of (hydroxy)chloroquine in patients with COVID-19 of any severity (15). This recommendation was informed by the second update of a living network meta-analysis that pooled data from 30 RCTs with 10,921 participants with COVID-19 and showed that hydroxychloroquine probably does not reduce mortality or mechanical ventilation (44).

Although the evidence on (hydroxy)chloroquine ± azithromycin was initially inconclusive, with only small RCTs and cohort studies of low-quality available, it became apparent over time that (hydroxy)chloroquine was not an effective treatment for patients hospitalized for COVID-19 and might even cause harm given the side effects. The strongest evidence for the lack of efficacy and safety of (hydroxy)chloroquine was provided by the large platform trials RECOVERY and SOLIDARITY.

## Discussion

The COVID-19 pandemic set of an unprecedented research endeavor to find treatment options for the disease. Investigators faced the challenge to balance scientific rigor required to set up controlled studies that provide reliable guidance with the urgency of responding to patients' immediate needs. In our analysis of the first year of the pandemic, we distinguished between the overall body of evidence for repurposed treatments and the uptake of clinical data as direct or indirect evidence in international and national treatment guidelines over time to identify the best approaches to sound evidence generation.

### Body of Evidence Evolving Over Time

The body of clinical evidence for corticosteroids, anticoagulants, and (hydroxy)chloroquine derived from a literature search was mainly informed by retrospective cohort studies, several, partly open-label RCTs, and few multi-arm platform trials that provided an increasing level of certainty as time progressed.

The first evidence available was from retrospective cohort studies. These studies are inexpensive to conduct and suitable for providing descriptive data on disease progression, risk factors, and treatments. However, they are inherently biased due to their observational nature. Although statistical methods to control confounding was applied in 85.4% of observational studies obtained from the literature search for corticosteroids, confounder control is still the weak point of observational studies as it depends on the quality of the data and whether the model has been correctly specified (84). Unmeasured confounders and residual confounding may lead to incorrect conclusions (85). Further points of criticism comprise poor quality of study reporting according to the guidelines of reporting observational studies [STROBE; (86)], or failure to include key clinical endpoints such as mortality or hospitalization duration as primary endpoints (87). Only large cohort studies using advanced methods to control for confounding were considered by experts and informed treatment guidelines (68, 80). Still, results from observational studies only have a low level of confidence and promising treatments need to be further investigated in RCTs.

Randomized clinical trials are considered the gold standard for demonstrating safety and efficacy of new treatments. Randomization into treatment groups and blinding of treatments prevent confounding and thus promote the validity and reliability of results (88). In practice, however, many RCTs on promising COVID-19 treatment regimens launched during the early pandemic show weaknesses that limit the quality of evidence (4, 89, 90). Several studies were underpowered with small patient numbers [e.g., (58, 75, 81)] or had to be stopped due to poor recruiting as incidences declined regionally [e.g., (24, 91)]. This problem was aggravated by a single center recruitment approach [e.g., (92)].

Coordinated efforts and analyses in the form of meta-analyses can provide robust evidence even with small RCTs. The meta-analyses for corticosteroids conducted by the REACT Working Group (32) is a good example: The WHO involved trial investigators at an early stage, so there was early communication and cooperation of experts from different disciplines and a high level of harmonization and standardization was achieved through this coordination. The protocol, data cut and publications were also arranged together in advance leading to joint and sound analyses and communication. All this led to corticosteroids being consistently recommended by the WHO, German, and US guidelines.

Adaptive platform trials offer flexible features, such as discarding treatments due to futility, declaring one or more treatments superior, or adding new treatments or patient groups to be tested (93). A Bayesian framework allows frequent looks into the data in context of interim analyses [e.g., multiplatform analysis for anticoagulation (94)]. As we show in our analysis, platform trials were a success story in the clinical research agenda of COVID-19, strongly impacting international treatment guidelines, while delivering fast and profound evidence (4, 95). The SOLIDARITY (83), RECOVERY (28, 82), and REMAP-CAP (23) platform trials were based on a blueprint or pre-existing protocol prepared by scientific networks (e.g., WHO R&D Blueprint group). They were able to start recruiting patients as early as March 2020 because the infrastructure and master protocol only needed to be adapted to the specific circumstances of the COVID-19 pandemic. The RECOVERY protocol was based on broad, simple inclusion criteria, central randomization, no additional biological samples or extraneous data collection, and the simple, unambiguous primary outcome of all-cause mortality (28). This pragmatic approach proved successful, as it took RECOVERY only 3 months to demonstrate that hydroxychloroquine offered no clinical benefit for COVID-19 patients (82), with the result that further clinical studies of (hydroxy)chloroquine were stopped [e.g., (96)]. The involvement of multiple countries in the SOLIDARITY platform trial allowed to shift recruitment to centers with high disease incidence (97). The REMAP-CAP protocol also allows to guide randomization based on data accumulated from patients already participating in the study (98). This adaptive approach increases the likelihood that patients are randomized to treatments that are more likely to be beneficial. These platform trials offered robust evidence that clearly demonstrated the superiority or futility of the treatments investigated. Yet, the innovative trial designs of platform trials, using Bayesian framework, can present new challenges to regulatory authorities and HTA bodies. The European Network for Health Technology Assessment (EUnetHTA) conducted rolling reviews for COVID-19 treatments and included the platform trials RECOVERY and REMAP-CAP in their assessment of corticosteroids (99).

### Evidence Supporting Treatment Recommendations During the Early Pandemic

As in clinical research, the development of treatment guidelines in the COVID-19 pandemic took an extraordinary course under intense time pressure. Within a year of the pandemic outbreak, treatment options such as corticosteroids or anticoagulants have been shown to be effective in treating COVID-19 disease and accompanying symptoms depending on the level of severity and thus were included in the treatment guidelines, based on clinical evidence of varying quality. Other treatment options that were considered during the early pandemic, such as (hydroxy)chloroquine, failed to prove efficacy and safety in COVID-19 and are therefore no longer used in patient care. This mix of positive and negative examples provides a comprehensive picture of the factors that promote or hinder high quality of evidence generation.

Despite the complexity of the development process, updates of treatment guidelines had to occur very quickly due to an urgent need from clinicians for evidence-based recommendations. The preparation process of treatment guidelines involves the review and evaluation of the available evidence, the derivation of recommendations, and a consensus voting of expert panels. In the first weeks of the COVID-19 pandemic, there were almost no studies on possible therapeutic options. Thus, the first versions of the guidelines had to rely on studies and guidelines of similar diseases or symptoms, such as MERS or ARDS, on *in-vitro* studies, or on evidence sources of lower quality, such as expert opinions, press releases of preliminary results from ongoing studies or pre-printed studies. Later, systematic analyses of evidence such as living reviews and meta-analyses facilitated the process. In order to adapt to this constantly changing evidence situation, the treatment guidelines for hospitalized patients were continuously revised resulting in 7, 5, and 21 versions from the WHO, Germany, and US, respectively. The expert panels proceeded in a very transparent manner and graduated the strength of the recommendation. The US guidelines, for example, provided a very well-founded, detailed overview of the underlying evidence base, its level of confidence as well as its limitations. The latest recommendations on treatment of hospitalized patients with COVID-19 were not in the scope of this analysis but can be found on following websites for the WHO (https://www.who.int/teams/health-care-readiness/covid-19), the AWMF/Germany (https://www.awmf.org/leitlinien/detail/ll/113-001LG.html), and the NIH/US (https://www.covid19treatmentguidelines.nih.gov/).

### Limitations

Limitations of our analysis may concern the literature search, which was conducted only in MEDLINE. Still, almost all published studies that were referred to in the guidelines were also found in the literature search. Although we may have missed a relevant publication, we assume that the selection of studies is representative in terms of design, patient population, and region for the studies conducted in the first year of the COVID-19 pandemic. A further challenge is related to the early versions of the guidelines where the consensus process is not fully clear. Additionally, the clinical data from which statements and recommendations were derived were not always explained in much detail and suffered in part from references to the literature difficult to follow [e.g., US guidelines, (22) February 11, 2021, referring to ASH frequently asked questions]. Finally, we were also confronted with the well-known publication bias, which most likely influences our analysis. Many completed studies had published their results only as preprints or have not yet been published at all (100).

### Future Directions

When we compare the overall body of evidence identified in our literature search with the studies directly or indirectly referenced in treatment guidelines for COVID-19, we found that clinical evidence from RCTs is included to a greater extent than observational data. This is not surprising, since RCTs are the most important source of information when the principles of evidence-based medicine are applied. Nevertheless, observational data played a substantial role informing treatment guidelines during the early phase of the pandemic, when data from patients treated with repurposed drugs were retrospectively analyzed in cohort studies. Observational studies are mainly incorporated into the guidelines by systematic reviews or clinical guidance documents provided by professional societies. To ensure a valid analysis, qualitative standards should be applied to observational studies:

- Sufficiently large sample size needed to draw generalizable conclusions; cooperation of several study centers might be beneficial here [e.g., Nadkarni et al. (68)].
- Protocol templates and technical infrastructure for data collection (e.g., electronic health records, data warehouse) and analyses to be able to start the study quickly [e.g., Geleris et al. (80)].
- Use of advanced statistical methods (e.g., propensity score methods, regression models) to control for confounding [e.g., Nadkarni et al. (68)].
- Publish results in form of a full study publication instead of letters and follow international reporting standards (e.g., STROBE) to provide all important information, so strengths and limitations can be determined [e.g., Geleris et al. (80)].

Randomized clinical trials, platform trials, and meta-analyses offered robust evidence for reliable treatment guidelines. We identified factors that led to rapid and robust evidence generation:

- Simple large trials with enough power to identify expected small effects with non-specific treatments (e.g., SOLIDARITY).
- Pre-existing research networks and international scientific organizations (e.g., REMAP-CAP, WHO R&D Blueprint group) provide an established infrastructure and pragmatic to adapt blueprints, and master-protocols.
- Multi-center trials active in various countries and continents provide a sufficient sample size, less dependent on the regional incidence (e.g., SOLIDARITY, RECOVERY).
- Standardized and harmonized protocols and common outcome measures (e.g., WHO ordinal clinical progression scale) ensure that data is shared and can be used in (pre-planned) meta-analyses (e.g., REACT Working Group).
- Adaptive study designs, ideally paired with a Bayesian framework, offer flexible tools to react dynamically to the pandemic situation, e.g., adding and dropping treatment arms, update allocation of randomization, or frequent and timely analyses (e.g., REMAP-CAP). Pragmatic elements such as the use of electronic health records (e.g., REMAP-CAP) and incorporation of assessment into clinic routines (e.g., SOLIDARITY) facilitate the participation of study centers.
- International reporting standards (e.g., CONSORT or PRISMA) should be followed to allow critical evaluation of strength and limitations and results should be provided in a timely manner in a peer-reviewed format.

### Conclusion

Pandemic preparedness, coordinated efforts, and combined analyses were crucial to generating robust clinical evidence that informed national and international treatment guidelines on corticosteroids, anticoagulation, and (hydroxy)chloroquine during the early COVID-19 pandemic. Multi-arm platform trials with master protocols and coordinated meta-analyses proved particularly successful, with researchers joining forces to answer the most pressing questions as quickly as possible. This was best achieved when networks and structures were already in place.

## Data Availability Statement

The original contributions presented in the study are included in the article/Supplementary Material, further inquiries can be directed to the corresponding author/s.

## Ethics Statement

Ethical review and approval was not required for the study on human participants in accordance with the local legislation and institutional requirements. Written informed consent for participation was not required for this study in accordance with the national legislation and the institutional requirements.

## Author Contributions

FL, SW, and SH were involved in the conception of this analysis. FL, KS, AL, DG-F, SW, and SH discussed and agreed on the methods used for the analysis. SW and SH conducted the search, extracted data, analyzed it, and wrote the initial draft of the manuscript. All authors revised the manuscript. All authors read and approved the final manuscript.

## Funding

Publication of this article was funded by Pfizer Deutschland GmbH, Novartis Pharma GmbH, and Amgen GmbH.

## Conflict of Interest

This study received funding from Pfizer Deutschland GmbH, Novartis Pharma GmbH, and Amgen GmbH. The funders had the following involvement with the study: FL (Pfizer) was involved in the conception of this analysis. FL (Pfizer), KS (Pfizer), AL (Amgen), DG-F (Novartis) discussed and agreed on the methods used for the analysis, revised the manuscript, and approved the final version. The remaining authors declare that the research was conducted in the absence of any commercial or financial relationships that could be construed as a potential conflict of interest.

## Publisher's Note

All claims expressed in this article are solely those of the authors and do not necessarily represent those of their affiliated organizations, or those of the publisher, the editors and the reviewers. Any product that may be evaluated in this article, or claim that may be made by its manufacturer, is not guaranteed or endorsed by the publisher.
