# Supplementary material for: Clinical Evidence Informing Treatment Guidelines on Repurposed Drugs for Hospitalized Patients During the Early COVID-19 Pandemic: Corticosteroids, Anticoagulants, (Hydroxy)chloroquine
Source: Front Public Health. 2022 Feb 18;10:804404. doi: 10.3389/fpubh.2022.804404 (PMC8896497; doi:10.3389/fpubh.2022.804404)
Supplement: Supplementary file 1 [file Data_Sheet_1.PDF]

Supplementary material file 1

Additional Table 1: Search strategy

Ovid MEDLINE(R) and Epub Ahead of Print, In-Process, In-Data-Review & Other Non-Indexed Citations, Daily and Versions(R)

#	search term	result
1	exp Coronavirus/	71150
2	exp Coronavirus Infections/	86638
3	(coronavirus* or corona virus* or OC43 or NL63 or 229E or HKU1 or HCoV* or ncov* or covid* or sars-cov* or sarscov* or Sars-coronavirus* or Severe Acute Respiratory Syndrome Coronavirus* or "Kawasaki like paediatric inflammatory multisystem syndrome" or "Kawasaki like pediatric inflammatory multisystem syndrome" or "PIMS-TS" or "Kawa-COVID-19" or "MIS-C" or "multisystem inflammatory syndrome in children" or pediatric multisystem inflammatory disease).mp.	148887
4	(or/1-3) and ((20191* or 202*).dp. or 20190101:20301231.(ep).)	135901
5	4 not (SARS or SARS-CoV or MERS or MERS-CoV or Middle East respiratory syndrome or camel* or dromedar* or equine or coronary or coronal or covidence* or covidien or influenza virus or HIV or bovine or calves or TGEV or feline or porcine or BCoV or PED or PEDV or PDCoV or FIPV or FCoV or SADS-CoV or canine or CCov or zoonotic or avian influenza or H1N1 or H5N1 or H5N6 or IBV or murine corona*).mp.	50920
6	((pneumonia or covid* or coronavirus* or corona virus* or ncov* or 2019-ncov or sars*).mp. or exp pneumonia/) and Wuhan.mp.	5099
7	(2019-ncov or ncov19 or ncov-19 or 2019-novel CoV or sars-cov2 or sars-cov-2 or sarscov2 or sarscov-2 or SARS-2-nCoV or SARS-2-Cov or SARS-COV-19 or Sars-coronavirus2 or Sars-coronavirus-2 or SARS 2 coronavirus* or Severe Acute Respiratory Syndrome-CoV-2 or SARS-like coronavirus* or coronavirus-19 or covid19 or covid-19 or covid 2019 or ((novel or new or nouveau) adj2 (CoV or nCoV or covid or coronavirus* or corona virus or Pandemi*2)) or ((covid or covid19 or covid-19 or SARS-CoV-2) and pandemic*2) or (coronavirus* and pneumonia)).mp.	133943

#	search term	result
8	(COVID-19 or SARS-CoV-2).rx,px,ox,rn. or (COVID-19 or COVID-19 serotherapy or ORF7b protein, SARS-CoV-2 or ORF6 protein, SARS-CoV-2 or ORF8 protein, SARS-CoV-2 or pediatric multisystem inflammatory disease, COVID-19 related or envelope protein, SARS-CoV-2 or ORF7a protein, SARS-CoV-2 or spike protein, SARS-CoV-2 or ORF3a protein, SARS-CoV-2 or COVID-19 drug treatment or severe acute respiratory syndrome coronavirus 2 or membrane protein, SARS-CoV-2 or ORF1ab polyprotein, SARS-CoV-2 or nucleocapsid protein, Coronavirus or COVID-19 vaccine or COVID-19 diagnostic testing).os,ps,rn,rs.	7448
9	("32185863" or "32172715" or "32227595" or "32140676" or "32246156" or "32267941" or "32176889" or "32169616" or "32265186" or "32253187" or "32152148" or "32053580" or "32179788" or "32213260" or "32205350" or "32188729" or "32152361" or "32277065" or "32088947" or "32240583" or "31917786" or "32127714" or "32047315" or "32020111" or "32240632" or "32243118" or "32267344" or "32239781" or "32396977" or "32402130" or "32243299" or "32807526" or "32344395" or "32403202" or "32389714" or "32416016" or "32405099" or "32976849" or "32685966" or "33221888" or "32379271" or "32188728" or "32221976" or "32417321" or "32489438" or "32332959" or "32943452" or "32807525" or "32826274" or "32898560" or "32293023" or "33159926" or "32919952" or "32835716" or "32619499" or "32663524" or "32392627" or "32392625" or "33037657" or "32777045" or "32521569" or "32492200" or "32930765" or "33075143" or "32237249" or "32683439" or "32495994" or "32344447" or "32896006" or "32240549" or "32438448" or "32425477" or "32951095" or "32274794" or "32750178" or "32463935" or "32428286" or "32491981" or "32930748" or "32119409" or "32432657" or "33003176" or "32459319" or "32822920" or "32878290" or "32270498" or "32250493" or "32512243" or "32837399" or "32426074" or "32199942" or "32839969" or "32639522" or "33073717" or "32502134" or "32334003" or "32510470" or "32819741" or "32309248" or "32243951" or "32378772" or "32835361" or "32962779" or "32916324" or "32785973" or "32272221" or "32299207" or "33044515" or	154

#	search term	result
	"33134955" or "32970917" or "32407438" or "32513790" or "32439468" or "33063036" or "33077677" or "32406056" or "32716821" or "32588590" or "32239757" or "32829902" or "32807521" or "32379350" or "33125767" or "32829731" or "32988821" or "32780977" or "32648633" or "32829907" or "32330635" or "32692998" or "33013067" or "33010706" or "32502292" or "32780969" or "32998780" or "32754731" or "32639607" or "32233030" or "32953429" or "32246897" or "32955802" or "32425490" or "32418270" or "32445255" or "32775945" or "32775948" or "32775953" or "32407043").ui.	
10	or/5-9	136585
11	exp glucocorticoids/ or exp prednisolone/ or exp Steroids/	900390
12	(corticosteroid* or glucocorticoid* or dexamethason* or prednisolon* or methylprednisolone or steroid* or hydrocortison*).mp	643806
13	11 or 12	1214533
14	exp Chloroquine/	18697
15	(chloroquin* or resochin* or hydroxychloroquin* or plaquenil*).mp	30757
16	14 or 15	30757
17	exp anticoagulants/ or exp heparin/	226887
18	(heparin* or anticoagula* or LMWH* or enoxaparin* or apixaban* or endoxaban* or rivaroxaban* or thromboprophyla* or VTE-prophyla*).mp	213291
19	17 or 18	306306
20	13 or 16 or 19	1532137
21	10 and 20	7980
22	(review* or news* or case* or editorial* or comment*).pt	6296563
23	21 not 22	4633
24	limit 23 to english language	4500
25	20200101:20210228.(dt).	1743165

#	search term	result
26	24 and 25	3731

Additional Table 2: PICO scheme

	Inclusion criteria	Exclusion criteria
Population	Hospitalized COVID-19 patients/SARS-CoV-2-positive adults	Healthy, children, pregnant women, outpatients<br>Pre-exposure prophylaxis<br>Subpopulations: cancer, transplant recipients, rheumatoid arthritis, asthma, inflammatory bowel disease, renal replacement
Intervention	Corticosteroids (e.g. dexamethasone, hydrocortisone, methylprednisolone)<br>OR<br>Anticoagulants (e.g. low-molecular weight heparin, fondaparinux)<br>OR<br>(Hydroxy)chloroquine	Other or no intervention<br>Intervention given for an underlying disease
Comparator	Any or none<br>(SOC, Placebo, other treatments, different dose of intervention)	Intervention in the background of an RCT with a different drug
Outcome	Mortality<br>Clinical status/ ventilator-free days<br>Hospitalization/ discharge<br>Symptom resolution<br>Viral clearance<br>(Serious) adverse events<br>Adverse events of special interest:	Biomarkers, pharmacokinetic, no results for population with an intervention of interest
Study type	Interventional or observational study, cohort study,	Case study, in vitro study
Publication type	Study publication, rapid/short communication, letter	Reviews, news, meta-analyses
Abbreviations: COVID-19: coronavirus disease 2019; RCT: randomized controlled trial; SARS-CoV-2: severe acute respiratory syndrome coronavirus type 2; SOC: standard of care		
